# The Annual Report of the Court of Directors of the Western Lunatic Asylum to the Legislature of Virginia, with the Report of the Superintendent and Physician

**Published:** 1843-04-15

**Authors:** 


### BIBLIOGRAPHICAL NOTICES.

*The Annual Report of the Court of Directors of the Western Lunatic Asylum to the Legislature of Virginia, with the Report of the Superintendent and Physician. For* 1842. Staunton: 1843. 8vo. pp. 61.

The perusal of this report has afforded us sincere gratification and pleasure. The extension of well managed institutions like the present, is to be desired by every friend of humanity.

One hundred and fifty-two insane persons have been accommodated with apartments in the Asylum during the year, of whom one hundred and five were males, and forty-seven females. The whole cost to the Commonwealth of this large number of patients, was $20,000, which sum, divided among the white population of the State, would give two cents and a half for each individual. The great disproportion between the sexes, is owing to the inability to accommodate, safely and conveniently, more than forty females. The number of female applicants on the list is above eighty; and additional buildings, designed for their especial use, are in course of construction, from legislative appropriation.

Table III confirms the general experience on the subject, that insanity is a much more common disease amongst the single, than the married portion of the community. From Table V. it would appear that few patients in the institution "became insane earlier than the age of puberty, or later than that period of life when the mental and physical energies usually begin to decline. A far greater number of the cases occurred between the ages of twenty and thirty-five, than within any similar period in point of duration. These facts correspond with what we see reported from most other institutions for the insane in this country, and tend strongly to prove that when the mental and physical powers are at their acme, and most actively employed, and at the period when the cares and responsibilities of life are usually most onerous, then is this sad affliction most to be dreaded."

Of the whole number of patients,

"But twenty-one had been insane less than one year, and but twenty-two between one and three years; whilst nineteen had been in that condition from three to five years, thirty-two from five to ten years, fifteen from ten to fifteen years, twenty-six from fifteen to twenty years, and six from twenty to thirty years. Those reported unknown, were for the most part, wanderers from other States, of whose history nothing could be ascertained—but the presumption is not unreasonable, that many of them had been so long burthensome to their friends, as to have reconciled them at least to their departure."

Dr. Stribling thinks that experience has amply demonstrated the fact, that but few persons afflicted with insanity for a longer period than two years, are ever restored to reason. Much, however, can be done to alleviate their sufferings, and render them contented and comfortable.

Dr. S. has found decided benefit to result from subjecting his patients to a thorough course of medical treatment. He regrets that so little of the general principles which should govern the treatment of this disease is known by the profession generally, and he thinks this want of information is frequently a serious evil. He says:

"It rarely happens that a patient is brought here, after having previously been under the care of a medical practitioner, in regard to whom it cannot be said that he has been '*well bled, blistered and purged.*' So indiscriminate and universal is this practice, and to such an extent is it frequently prosecuted, that it numbers amongst its *victims*, those labouring under every form, degree, and duration of insanity! But those who are most exposed to it, and in the greatest degree injured by it, are individuals afflicted with active mania. Here the practitioner rarely fails to attribute the usual consequences, which result alone from *nervous* excitement, to inordinate *arterial* action. The incessant ravings, extreme restlessness, flushed countenance, heat of skin, excited pulse, distended blood vessels, and almost supernatural muscular power, which in such cases are but *effects*, are unfortunately, for want of proper discrimination, viewed as *symptoms* of the disease, and the unhappy patient is forthwith subjected to a course of *active depletion*, with *blisters*, designed to act as revulsives, which invariably increase the evils they were intended to relieve. Whereas, had he been placed as far as practicable beyond the influence of external excitants, the circulation equalized by cold applications to head, with the warm foot bath, and after a gentle laxative, for the purpose of removing whatever by its presence in the intestinal canal might have proved irritating, been subjected to the influence of narcotics, administered freely and at short intervals, the result must have been far different. I would by no means assert that cases never occur, in which a free use of the lancet, and other depletory measures, are not absolutely required, for the reverse is the fact; but such cases are, beyond question, *comparatively* rare, whilst the practice complained of, is, as before remarked, almost universal."

The highest degree of nervous excitement is compatible with an entire absence of any circulatory disturbance, and yields speedily to a treatment which would quickly aggravate a similar case, dependent on vascular excitement. Suitable moral means, in the treatment of insanity, is of the first importance, and seems to be adequately appreciated in the Staunton establishment. A system of classification is pursued, which, besides securing good order and the appearance of comfort, operates powerfully in promoting good deportment, from the dread of being degraded to a lower class. Promotion to a higher class is attended with corresponding results. At the table order is preserved; and one hundred and twenty-nine patients eat more or less regularly at the tables. Amusements of various kinds are furnished, and are engaged in by the patients with good effects. Exercise in the open air, in suitable weather, is freely taken.

"Having experienced great difficulty in inducing the third class of male patients to take sufficient exercise, owing to their mental and physical energies being almost prostrated by the duration of their disease, the experiment was adopted in the course of the year of organizing them into a militia company, officered from amongst themselves, and supplied with badges to designate their appropriate rank. In suitable weather they paraded frequently, and by the tap of the drum were marched under the supervision of one or more of the attendants for several miles into the country. Besides the good effects of fresh air and wholesome exercise in invigorating their physical health and reviving their spirits, many were induced to manifest an interest as to the dress, order, &c., who had previously seemed indifferent to every means resorted to for the purpose of amusing or occupying them. This company has been thrown somewhat into disorder, in consequence of the severity of the weather having interrupted the parades—but chiefly because its captain has recovered his reason and left us, to engage in more profitable employment; there are, however, several aspirants for the office thus vacated, and we anticipate, so soon as the spring opens, reviving our military spirit, re-organizing our forces, and unfolding again our colours to the breeze."

Music constitutes a favourite recreation; and performances on the harp, piano, guitar and flute, constantly enliven the household.

Books, of a light and amusing character, are eagerly sought after by many of the inmates, and with some, historical works are a constant subject of study. The books are well kept, and returned in good order. The largest class of readers, however, prefer the newspapers, and those with matters of local interest are the chief favourites.

With respect to labour and employment, the Report says:

"Each year's experience tends but the more strongly to impress us with a conviction that of all the moral means resorted to for the benefit of the insane, none can compare in degree or extent of usefulness with appropriate manual labour."

The institution, too, is largely benefitted by this system. The produce of the farm and garden alone were worth $980 for the past year; and the female patients produced by their work $463.

The importance of sending the insane early to an asylum is strongly urged. Many are deterred from so doing from ignorance of the actual condition of Institutions of this nature.

The report terminates with some well written remarks on simulated insanity.

With this imperfect notice of Dr. Stribling's labours we take our leave of them, with an acknowledgement of the pleasure we have derived from their perusal; and venture to express the hope that his philanthropic views may be fully carried out, and crowned with the success they deserve.

